# Cost-effectiveness analysis of mesh fixation techniques for laparoscopic and open inguinal hernia surgeries

**DOI:** 10.1186/s12913-022-08491-4

**Published:** 2022-09-06

**Authors:** Suphakarn Techapongsatorn, Amarit Tansawet, Oraluck Pattanaprateep, John Attia, Gareth J. Mckay, Ammarin Thakkinstian

**Affiliations:** 1 Department of Clinical Epidemiology and Biostatistics, Faculty of Medicine Ramathibodi Hospital, Mahidol University, Bangkok, Thailand; 2 Department of Surgery, Faculty of Medicine Vajira Hospital, Navamindradhiraj University, Bangkok, Thailand; 3 School of Medicine and Public Health, and Hunter Medical Research Institute, University of Newcastle, New Lambton, NSW Australia; 4 Center for Public Health, School of Medicine, Dentistry and Biomedical Sciences, Queen’s University Belfast, Belfast, United Kingdom

**Keywords:** Inguinal hernia, Mesh fixation, Cost-effectiveness analysis

## Abstract

**Purpose:**

This study reports economic evaluation of mesh fixation in open and laparoscopic hernia repair from a prospective real-world cohort study, using cost-effectiveness analysis (CEA) and cost-utility analysis (CUA).

**Methods:**

A prospective real-world cohort study was conducted in two university-based hospitals in Thailand from November 2018 to 2019. Patient data on hernia features, operative approaches, clinical outcomes, associated cost data, and quality of life were collected. Models were used to determine each group’s treatment effect, potential outcome means, and average treatment effects. An incremental cost-effectiveness ratio was used to evaluate the incremental risk of hernia recurrences.

**Results:**

The 261 patients in this study were divided into six groups: laparoscopic with tack (LT, *n* = 47), glue (LG, *n* = 26), and self-gripping mesh (LSG, *n* = 30), and open with suture (OS, *n* = 117), glue (OG, *n *= 18), and self-gripping mesh (OSG, *n* = 23). Hernia recurrence was most common in LSG. The mean utility score was highest in OG and OSG (both 0.99). Treatment costs were generally higher for laparoscopic than open procedures. The cost-effectiveness plane for utility and hernia recurrence identified LSG as least cost effective. Cost-effectiveness acceptability curves identified OG as having the highest probability of being cost effective at willingness to pay levels between $0 and $3,300, followed by OSG.

**Conclusion:**

Given the similarity of hernia recurrence among all major procedures, the cost of surgery may impact the decision. According to our findings, open hernia repair with adhesive or self-gripping mesh appears most cost-effective.

**Supplementary Information:**

The online version contains supplementary material available at 10.1186/s12913-022-08491-4.

## Background

Inguinal hernia is a common global condition, resulting in more than 20 million surgical repairs annually [1]. A hernia repair with mesh graft is considered the standard of care for adult symptomatic patients following the international guidelines for groin hernia management [1] and can be performed using an open or laparoscopic approach. The use of mesh repair strengthens the inguinal floor, reducing the risk of hernia recurrence due to its proximity to the herniated triangle. Several surgical interventions are commonly used, namely, open inguinal hernia repair [(OHR; i.e., Lichtenstein’s repair] and laparoscopic inguinal hernia repair [(LHR) i.e., transabdominal preperitoneal repair (TAPP) and total extra-peritoneal repair (TEP)]. According to recent inguinal hernia repair guidelines, mesh fixations are recommended in patients with large direct hernias (M3-EHS classification) [1]. For OHR and LHR, suture and tack are commonly used for mesh fixation, respectively. As alternatives, non-penetrating or atraumatic fixation techniques have been proposed, including glue and self-gripping mesh (SGM).

However, complications following mesh fixation include local tissue trauma and nerve damage through entrapment, [2] erosion, [3] meshoma formation, [4] tack hernias, [5] chronic pain, [6, 7] and infection [8]. Various mesh fixation methods have been developed for open and laparoscopic approaches, such as tack, glue, and self-gripping mesh (SGM). The efficacy and adverse effects for open and laparoscopic mesh fixation methods have been compared using multiple meta-analyses, [9–19] which were summarized in an umbrella review indicating that various options are largely similar in terms of hernia recurrence and adverse events; therefore, the technique of choice may be influenced by other considerations such as cost. Therefore, we conducted an economic evaluation of real-world data from a prospective cohort to evaluate both cost-effectiveness (CEA) and cost-utility (CUA) of mesh fixation in open and laparoscopic inguinal hernia repair.

## Methods

### Study sites and design

A 12-month prospective cohort study was conducted at the tertiary referral centers of Ramathibodi and Vajira Hospitals, Bangkok, Thailand, following the methods for the economic evaluation of health care programmes [20]. Patients were eligible if aged > 18 years, were diagnosed with primary uncomplicated inguinal hernia, underwent OHR and LHR with any mesh fixation approach, had a pre-anesthesia ASA Physical Status Classification of I–III, and were not immunocompromised. This study was approved by the Vajira Institutional Review Board (COA No. 160/2561) and the Committee on Human Rights Related to Research Involving Human Subjects, Ramathibodi Hospital (COA No. MURA2018/986).

### Interventions of interest

Patients were divided into six intervention groups based on the actual received operative and mesh fixation techniques: LHR-tack (LT, reference), LHR-glue (LG), LHR-self-gripping mesh (LSG), OHR-suture (OS, reference), OHR-glue (OG), and OHR-self-gripping mesh (OSG). The selected techniques were based on discussion and decision between surgeons and patients without researcher manipulation. In LT group, the metallic tack was used for mesh fixation with 3 locations, i.e., pubic symphysis, anterior abdominal wall medial, and lateral to the epigastric vessels. Data on age, sex, body mass index (BMI), underlying disease (e.g., diabetes, hypertension, joint disease, benign prostatic hyperplasia, chronic pulmonary disease, and obesity), and potentially risky patient behaviors such as weight-lifting activities/exercise or constipation were collected.

### Cost of illness data

The study evaluated individual patient-level data associated with hernia treatment expenditure. From a hospital perspective, direct medical costs were retrieved from hospital invoices including room fee, operative, anesthetic, laboratory, medical, and imaging charges. From a societal perspective, direct nonmedical and indirect costs were also collected through patient and caregiver interviews. Direct nonmedical costs included transportation, accommodation, and extra-meal fees, whereas indirect costs included patient and caregiver productivity loss calculated from days lost from work multiplied by salary.

Data were collected through patient interviews at three time points: preoperatively and postoperatively for the short term (discharge day, 1–2 weeks, 1 month) and long-term (6 months). Patients were followed up after discharge at out-patient clinic or by telephone, where appropriated. The time horizon for the utility was defined as short term (1-week postoperatively) and long-term (6 months postoperatively); hernia recurrence was assessed at study termination. The cost estimation for hernia recurrence was considered based on the OHR and LHR conventional mesh fixation. Due to the short horizon time, discount rates were not applied in the analyses.

All currencies were obtained in Thai Baht and converted to US dollars (USD) using the Bank of Thailand exchange rate on December 30, 2020 (30.014 Baht/US Dollar) [21].

### Clinical and utility outcomes

The primary clinical outcome was hernia recurrence evaluated through physical examination and/or radiographic investigation. Secondary outcomes included postoperative pain measured using the visual analog score (VAS), hematoma, urine retention, surgical site infection (SSI), and seroma. The utility outcome was determined using the Thai EQ-5D-5L [22] and Euro-Quality of life visual analog scale (EQ-VAS) questionnaires [22]. The EQ-5D-5L questionnaires were previously validated in a Thai population, assessing five health dimensions: mobility, self-care, usual activities, pain/discomfort, and anxiety/depression. Responses generated from the EQ-5D-5L questionnaires were converted into a utility score based on a Thai reference set; the utility outcome determined using the Thai EQ-5D-5L was developed by a research team from the Health Intervention and Technology Assessment program, Ministry of Public Health, Thailand [23, 24]. The utility outcome yields an index score anchored at 0 (dead) and 1 (full health). All outcomes were collected 1 week preoperatively and 1 month and 6 months postoperatively.

### Statistical analysis

Data were described using frequency and mean (standard deviation [SD]) for categorical and continuous data, respectively. Patient baseline characteristics (age, sex, BMI, and underlying disease status) and baseline utility scores were compared among the six intervention groups using Chi-squared (or exact test where appropriate) and one-way analysis of variance/regression for categorical and continuous data, respectively. Variables with a *p*-value < 0.1 were considered further for inclusion within adjusted regression models.

Intervention effects were assessed using the following approaches: First, a multilevel mixed-effects linear regression model assessed the treatment effects by fitting intervention groups against utility scores (measured at discharge day, 1 week, and 6 months postoperatively) with adjustment for covariables identified from the univariate analysis. Coefficients (i.e., mean difference [MD]) with 95% confidence interval (CIs) were estimated and tested. Second, the treatment effect was considered with adjustment of treatment assignment based on the following steps: the treatment model was established using a multi-logit model to estimate the probability of receiving each treatment by fitting covariables to the treatment variable. An outcome (utility score) model was created using an inverse-probability-weighted regression adjustment. Covariables in both the treatment and outcome models included age, sex, BMI, weight-lifting activities/exercise, and underlying diseases (e.g., diabetes, hypertension, cardiovascular disease, chronic pulmonary disease, urination difficulties, and constipation). Only significant covariables were retained within the final models. The potential outcome means (POMs) and average treatment effects (ATEs) were estimated with 95% CIs. Finally, the treatment effect model assumptions were evaluated to balance the distribution of covariables and treatment overlap.

An incremental cost-effectiveness ratio (ICER) was calculated by dividing the incremental cost with the incremental effect using tack as the reference for LHR and sutures as the reference for OHR. We used both short- and long-term time horizons for the utility outcome; however, only the long-term time horizon was used for hernia recurrence.

To evaluate the models’ robustness and the effects of parameter uncertainty, Monte-Carlo bootstrapping (× 1,000) was simulated to plot cost-effectiveness acceptability curves for six interventions using hospital and societal perspectives. All clinical and utility outcomes were analyzed using STATA version 16.1, and CUA was performed using MS Excel 2016 and TreeAge Pro 2020 [25].

## Results

### Patient characteristics

A total of 261 patients were included during the study period. Demographic and baseline characteristics of patients for each group are described and compared with intervention groups (Table 1). The mean age was 64.41 (SD: 14.24) years, 245 patients (93.87%) were male, and the mean BMI was 23.47 (SD: 3.40) kg/m^2^. Only age and constipation rate significantly varied among intervention groups.

**Table 1:** Baseline patient characteristics

Characteristics	Overall	LT	LG	LSG	OS	OG	OSG	*p-value*
Number of patients	261	47	26	30	117	18	23	
Age, years, mean (SD)	64.4 (14.2)	62.7 (15.8)	61.8 (16.8)	66.5 (11.2)	66.0 (12.7)	56.0 (19.7)	66.4 (11.9)	0.017
Male, number (%)	245 (93.9)	43 (91.5)	23 (88.5)	28 (93.3)	112 (96.6)	18 (100.0)	21 (91.3)	0.558
BMI, kg/m^2^, mean (SD)	23.5 (3.4)	23.5 (3.4)	22.6 (3.2)	23.6 (3.0)	23.34 (3.23)	22.9 (3.6)	25.2 (4.4)	0.491
Comorbidity, number (%)								
-Diabetes	33 (12.6)	6 (12.8)	2 (7.7)	3 (10.0)	16 (13.7)	3 (16.7)	3 (13.0)	0.950
-Hypertension	104 (39.8)	19 (40.4)	11 (42.3)	14 (46.7)	46 (39.3)	4 (22.2)	10 (43.5)	0.680
-Chronic kidney disease	6 (2.3)	0 (0.0)	0 (0.0)	1 (3.3)	3 (2.6)	1 (5.6)	1 (4.3)	0.673
-Cardiovascular disease	30 (11.5)	4 (8.5)	0 (0.0)	5 (16.7)	17 (14.5)	1 (5.6)	3 (13.0)	0.277
-Chronic pulmonary disease	24 (9.2)	2 (4.3)	3 (11.5)	4 (13.3)	11 (9.4)	3 (16.7)	1 (4.3)	0.547
-Chronic urinary problem	54 (20.7)	9 (19.1)	9 (34.6)	6 (20.0)	21 (17.9)	5 (27.8)	4 (17.4)	0.495
-Weight lifting activities	17 (6.5)	3 (6.4)	3 (11.5)	1 (3.3)	5 (4.3)	1 (5.6)	4 (17.4)	0.218
-Constipation	31 (11.9)	7 (14.9)	9 (34.6)	0 (0.0)	6 (5.1)	4 (22.2)	5 (21.7)	< 0.001

*LT* Laparoscopic inguinal hernia repair using tacker, *LG* Laparoscopic inguinal hernia repair using glue, *LSG* Laparoscopic inguinal hernia repair using self-gripping mesh, *OS* Open inguinal hernia repair using suture, *OG* Open inguinal hernia repair using glue, *OSG* Open inguinal hernia repair using self-gripping mesh

### Hernia data and operative outcome

There was a total of 245 and 16 patients with unilateral and bilateral inguinal hernias, respectively, including 212, 15, 2, and 49 indirect, direct, femoral, and combined hernias, respectively. Of the 103 LHR hernia repairs, 47, 26, and 30 patients received LT, LG, and LSG, respectively. OHR was performed in 148 patients; 117, 18, and 23 patients received OS, OG, and OSG, respectively.

The overall mean operative time and estimated blood loss were 75.42 (SD: 32.40) min and 10.74 (SD: 13.90) ml, respectively. The mean overall hospital stay was 3.82 (SD: 1.40) days, with a mean postoperative hospital stay of 2.63 (SD: 1.20) days. The mean postoperative pain VAS scores at 4 and 24 h were 6.75 (SD: 1.70) and 3.54 (SD: 1.60), respectively.

Individual postoperative complications (such as urinary retention, wound seroma, hematoma, and SSI) were minimal and were therefore combined into a composite complication endpoint. Hernia recurrence occurred in 4.60% (95% CI: 2.39, 7.89), chronic groin pain at 6 months was reported in 9.96% (95% CI: 6.61, 14.25), and composite complications were reported in 27.6% (95% CI: 22.25, 33.43) of the study cohort (see Table 2).

**Table 2:** Clinical and utility outcomes among 6 intervention groups

Characteristics	Overall	LT	LG	LSG	OS	OG	OSG	*p-value*
Number of patients	261	47	26	30	117	18	23	
Clinical outcome, mean (SD)								
-Operative time, min	75.42 (32.42)	83.40 (34.59)	61.73 (12.64)	102.17 (40.87)	74.50 (29.05)	63.89 (15.10)	53.35 (30.90)	< 0.001
-Estimated blood loss, ml	10.74 (13.92)	8.36 (7.95)	11.35 (18.62)	18.50 (25.12)	10.56 (12.02)	8.67 (4.99)	7.30 (6.37)	< 0.001
-Total hospital stays, day	3.82 (1.40)	4.34 (1.82)	4.46 (1.58)	3.53 (1.20)	3.50 (1.23)	4.00 (0.97)	3.91 (1.04)	0.002
-Post-operative stay, day	2.63 (1.23)	2.91 (1.21)	3.12 (1.28)	2.53 (1.20)	2.45 (1.35)	2.56 (0.70)	2.57 (0.66)	0.001
-Pain VAS at 4-h	6.75 (1.67)	6.06 (1.19)	6.81 (2.08)	6.83 (1.46)	7.15 (1.53)	6.67 (1.75)	5.96 (2.20)	0.004
-Pain VAS at 24-h	3.54 (1.55)	2.70 (1.16)	2.85 (1.35)	4.37 (1.50)	4.22 (1.18)	3.17 (1.42)	1.83 (1.75)	0.097
No. of events, n (%)								
-Recurrence	12 (4.60)	1 (2.13)	1 (3.85)	3 (10.00)	4 (3.42)	1 (5.56)	2 (8.70)	0.001
-Chronic groin pain	26 (9.96)	4 (8.51)	3 (11.54)	1 (3.33)	12 (10.26)	4 (22.22)	2 (8.70)	< 0.001
-Composite complication	72 (27.59)	15 (31.91)	7 (26.92)	7 (23.33)	25 (21.37)	8 (44.44)	10 (43.48)	< 0.001
-Wound complication	60 (22.99)	12 (25.53)	6 (23.08)	5 (16.67)	22 (18.80)	7 (38.89)	8 (34.72)	< 0.001
Utility index, mean (SD)								
-Pre-operation	0.94 (0.08)	0.94 (0.09)	0.91 (0.10)	0.96 (0.05)	0.96 (0.07)	0.93 (0.67)	0.88 (0.12)	< 0.001
-Post-operation	0.90 (0.09)	0.92 (0.07)	0.84 (0.09)	0.93 (0.10)	0.92 (0.08)	0.84 (0.08)	0.81 (0.12)	0.034
-At 1 week	0.97 (0.06)	0.97 (0.05)	0.95 (0.07)	0.98 (0.04)	0.98 (0.05)	0.96 (0.05)	0.93 (0.08)	0.002
-At 1 month	0.99 (0.02)	0.99 (0.03)	0.99 (0.04)	0.99 (0.02)	0.99 (0.02)	0.99 (0.01)	0.99 (0.02)	< 0.001
-At 6 months	0.99 (0.03)	0.99 (0.04)	0.99 (0.02)	0.99 (0.01)	0.99 (0.02)	0.99 (0.03)	0.99 (0.03)	< 0.001
EQ-VAS, mean (SD)								
-Pre-operation	75.69 (11.72)	79.15 (12.52)	75.19 (12.84)	70.33 (12.45)	75.68 (9.21)	78.61 (12.81)	73.91 (15.95)	0.003
-Post-operation	74.63 (12.79)	74.45 (11.14)	70.96 (14.56)	76.33 (10.58)	76.88 (10.94)	70.28 (12.89)	68.91 (20.72)	< 0.001
-At 1 week	81.40 (11.51)	80.96 (10.56)	81.35 (12.85)	81.33 (13.83)	83.07 (9.42)	80.00 (11.63)	75.00 (16.17)	0.003
-At 1 month	87.85 (8.78)	85.64 (10.61)	88.65 (9.75)	88.83 (7.51)	88.87 (7.94)	85.83 (7.33)	86.52 (9.82)	0.098
-At 6 months	93.46 (7.97)	90.64 (8.76)	93.08 (6.64)	98.33 (4.01)	94.53 (8.10)	88.33 (8.57)	91.82 (5.88)	< 0.001

^*^VAS Visual analog scale, *LT* Laparoscopic inguinal hernia repair using tacker, LG Laparoscopic inguinal hernia repair using glue, *LSG* Laparoscopic inguinal hernia repair using self-gripping mesh, *OS* Open inguinal hernia repair using suture, *OG* Open inguinal hernia repair using glue, *OSG* Open inguinal hernia repair using self-gripping mesh

### Utility outcome

The overall mean utilities of patients at preoperation, discharge, and postoperative 1 week, 1 month, and 6 months were 0.94 (SD: 0.08), 0.90 (SD: 0.09), 0.97 (SD: 0.06), 0.99 (SD: 0.02), and 0.99 (SD: 0.03), respectively; this is consistent with minor surgery and not really impacting perfect health (utility score of 1). The mean corresponding EQ-VAS scores for these time points were 75.69 (SD: 11.72), 74.63 (SD: 12.79), 81.40 (SD: 11.51), 87.85 (SD: 8.78), and 93.46 (SD: 7.97). The mean utility scores and EQ-VAS by intervention group are also provided (Table 2).

The overall mean utility was compared among intervention groups using a multilevel mixed-effects linear regression model adjusted for age and constipation at baseline. The overall mean utility scores were 0.966, 0.947, 0.972, 0.970, 0.945, and 0.939 for LT, LG, LSG, OS, OG, and OSG, respectively, with only LG, OG, and OS groups being significantly different to LT with MD of − 0.019 (95%CI: − 0.033, − 0.004), − 0.021 (95%CI: − 0.037, − 0.004), and − 0.026 (95%CI: − 0.041, − 0.011), respectively (Supplementary Table 1). Furthermore, linear comparisons of the overall mean utility scores among groups indicated MDs of − 0.002 (95%CI: − 0.020, 0.016) for OG versus LG and − 0.033 (95%CI: − 0.049, − 0.016) for OSG versus LSG.

A treatment effect model compared overall utility scores among six interventions. The balance of covariables among each of the intervention groups was assessed by exploring the distribution of covariables across all six interventions. Raw and standardized MDs and variance ratios for covariables were calculated based on intervention. After adjusting for covariables in the treatment effect models, standardized MDs were reduced to near zero, with variance ratios close to one (except for LSG with SMD of − 0.505, and variance ratio of zero) representing a reasonable balance across groups (Supplementary Table 2 and Supplementary Figs. 1 and 2).

The following POMs were estimated: LT: 0.966 (95%CI: 0.959, 0.973), LG: 0.951 (95%CI: 0.938, 0.964), LSG: 0.970 (95%CI: 0.956, 0.985), OS: 0.969 (95%CI: 0.964, 0.975), OG: 0.941 (95%CI: 0.927, 0.955), and OSG: 0.932 (95%CI: 0.913, 0.951) (see Supplement Table 3). The ATEs were also estimated: LSG: 0.004 (95%CI: − 0.012, 0.019), OS: 0.04 (95%CI: − 0.004, 0.011), LG: − 0.015 (95%CI: − 0.029, − 0.0004), OG: − 0.025 (95%CI: − 0.040, − 0.010), and OSG: − 0.334 (95%CI: − 0.054, − 0.014) relative to LT, with the latter three indicating significantly lower utility scores.

**Table 3:** Cost data among 6 intervention groups

Characteristics	Overall	LT	LG	LSG	OS	OG	OSG	*p-value*
Hospital perspective								
-Short term cost	1,318.41 (2,135.19)	1,637.01 (717.70)	1,427.71 (440.24)	2,498.98 (428.72)	1,036.78 (3,038.18)	682.36 (284.57)	934.40 (436.54)	< 0.001
-Long term cost	1,350.82 (2,144.81)	1,655.45 (729.47)	1,441.10 (453.33)	2,534.6 (454.02)	1,084.65 (3,062.71)	700.49 (301.46)	945.05 (452.50)	< 0.001
-Recurrence cost	1,245.64 (739.59)	1,509.05	1,099.19	2,345.41 (251.94)	572.42 (141.89)	720.13	1,146.70 (41.03)	< 0.001
-No recurrence cost	1,355.89 (2,190.43)	1,658.63 (737.20)	1,454.78 (457.17)	2,555.68 (469.51)	1,102.78 (3,115.28)	699.33 (310.70)	925.84 (469.79)	< 0.001
Societal perspective								
-Short term cost	1,888.07 (2,233.94)	2,379.29 (1,202.31)	1,728.19 (508.40)	3,222.23 (609.70)	1,612.01 (3,059.36)	965.88 (402.42)	1,450.83 (1,067.87)	< 0.001
-Long term cost	2,106.51 (2,307.87)	2,692.35 (1,433.61)	1,877.79 (551.42)	3,484.10 (738.54)	1,824.30 (3,116.30)	1,120.96 (520.44)	1,577.93 (1,130.15)	< 0.001
-Recurrence cost	2,233.39 (982.82)	2,263.69	1,584.96	3,593.71 (68.38)	1,749.83 (762.59)	881.72	2,144.90 (347.23)	< 0.001
-No recurrence cost	2,100.39 (2,353.79)	2,701.67 (1,448.01)	1,889.50 (559.48)	3,471.92 (778.77)	1,826.94 (3,168.97)	1,135.03 (532.92)	1,523.93 (1,167.79)	< 0.001

Mean values (standard edviation) costs in US dollars

*LT* Laparoscopic inguinal hernia repair using tacker, *LG* Laparoscopic inguinal hernia repair using glue, *LSG* Laparoscopic inguinal hernia repair using self-gripping mesh, *OS* Open inguinal hernia repair using suture, *OG* Open inguinal hernia repair using glue, *OSG* Open inguinal hernia repair using self-gripping mesh

**Table 4:** Cost-utility analysis among 6 intervention groups

	**Short term (1 week)**			**Long term (6 months)**		
	**Cost ($)**	**Incremental utility**	**ICER**	**Cost ($)**	**Incremental utility**	**ICER**
**Hospital perspective**						
LT	1,637.01	0.0278		1,655.45	0.0524	
LG	1,427.71	0.0390	cost saving	1,441.10	0.0813	cost saving
LSG	2,498.98	0.0195	dominated	2,534.65	0.0347	dominated
OS	1,036.78	0.0184		1,084.65	0.0341	
OG	682.36	0.0246	cost saving	700.49	0.0542	cost saving
OSG	934.40	0.0445	cost saving	945.05	0.1068	cost saving
**Societal perspective**						
LT	2,379.29	0.0278		2,692.35	0.0524	
LG	1,728.19	0.0390	cost saving	1,877.79	0.0813	cost saving
LSG	3,222.23	0.0195	dominated	3,484.10	0.0347	dominated
OS	1,612.01	0.0184		1,824.30	0.0341	
OG	965.88	0.0246	cost saving	1,120.96	0.0542	cost saving
OSG	1,450.83	0.0445	cost saving	1,577.93	0.1068	cost saving

*LT* Laparoscopic inguinal hernia repair using tacker, *LG* Laparoscopic inguinal hernia repair using glue, *LSG* Laparoscopic inguinal hernia repair using self-gripping mesh, *OS* Open inguinal hernia repair using suture, *OG* Open inguinal hernia repair using glue, *OSG* Open inguinal hernia repair using self-gripping mesh. *ICER* Incremental cost-effectiveness ratio. The difference in cost between glue or SGM mesh fixation compared to convention (tacker in LHR or suture in OHR groups), divided by the difference in their incremental utility. The cost saving means an incremental cost or utility is better than conventional group

**Table 5:** Cost-effectiveness analysis on hernia recurrence among 6 intervention groups

	**Cost ($)**	**Individual data from cohort study**			**Umbrella review**		
		**Recurrence rate**	**Incremental recurrence case prevented**	**ICER**	**Recurrence rate**	**Incremental recurrence case prevented**	**ICER**
**Hospital perspective**							
LT	2,761.41	2.13			2.36		
LG	2,557.56	3.85	-1.72	118.52	2.00	0.36	cost saving
LSG	3,658.46	10.00	-7.87	dominated	1.40	0.96	934.43
OS	2,761.41	3.42			2.68		
OG	2,357.96	5.56	-2.14	188.53	2.00	0.68	cost saving
OSG	2,584.48	8.70	-5.28	33.51	1.60	1.08	cost saving
**Societal perspective**							
LT	4,090.63	2.13			2.36		
LG	3,411.90	3.85	-1.72	394.61	2.00	0.36	cost saving
LSG	5,420.65	10.00	-7.87	dominated	1.40	0.96	1,385.44
OS	4,451.50	3.42			2.68		
OG	3,583.39	5.56	-2.14	405.66	2.00	0.68	cost saving
OSG	4,846.57	8.70	-5.28	dominated	1.60	1.08	365.81

*LT* Laparoscopic inguinal hernia repair using tacker, *LG* Laparoscopic inguinal hernia repair using glue, *LSG* Laparoscopic inguinal hernia repair using self-gripping mesh, *OS* Open inguinal hernia repair using suture, *OG* Open inguinal hernia repair using glue, *OSG* Open inguinal hernia repair using self-gripping mesh. The difference in cost between glue or SGM mesh fixation compared to convention (tacker in LHR or suture in OHR groups), divided by the difference in their incremental hernia recurrence rate. The cost saving means an incremental cost or utility is better than conventional group

### Cost outcomes

From a hospital perspective, the mean costs for short- and long-term outcomes were $1,318.41 (SD: 2,135.19) and $1,350.82 (SD: 2,144.81), respectively. From a societal perspective, the mean costs for short- and long-term outcomes were $1,888.07 (SD: 2,233.94) and $2,106.51 (SD: 2,307.87), respectively. The mean hospital costs associated with hernia recurrence (before reoperation) were $1,245.64 (SD: 739.59) and $1,355.89 (SD: 2,190.43) in those without recurrence. The mean societal costs associated with hernia recurrence (before reoperation) were $2,233.39 (SD: 982.82) and $2,100.39 (SD: 2,353.79) in those without recurrence. Treatment costs significantly varied among groups, with laparoscopic procedures being more expensive than open surgery. All cost data for each group are presented in Table 3.

### Economic evaluation outcomes

An individual economic data analysis model of hernia repair costs was performed for both OHR and LHR mesh fixation methods to compare incremental mean utility and recurrent case prevention. All costs included hospital and societal perspectives for short- and long-term outcomes for each intervention group. The mean costs associated with LHR were higher than those with OHR for both hospital and societal perspectives. LSG had the highest associated total costs, followed by LT, LG, OS, and OSG, with OG representing the least costly alternative (Table 3).

Incremental utility scores were highest for OSG, followed by LG, LT, OG, LSG, and OS. The ICER for short- and long-term outcomes were calculated for both hospital and societal perspectives. LG provided the highest cost savings in the laparoscopic surgical group, whereas OSG provided the best ICER for the open surgical group for both short- and long-term follow-up outcomes (Tables 4 and 5).

The cost-effectiveness plane was considered under two scenarios: utility and hernia recurrence prevention. The utility cost-effectiveness plane indicated that LSG was less cost effective from both hospital and societal perspectives and short- and long-term follow-up outcomes. LG, OG, and OSG provided better cost-effectiveness planes compared to LT and OS (Supplementary Fig. 3).

The cost-effective plane for the prevention of hernia recurrence indicated that LSG was less cost effective from both hospital and societal perspectives; OSG demonstrated less cost-effectiveness from the societal perspective. LG and OG both indicated less cost-effectiveness representing lower costs – less costly but with a higher hernia recurrence rate. The results demonstrated that LG, OG, and OSG dominated results, higher than that of LT and OS, respectively. Although LSG more effectively prevented hernia recurrence, it was more costly than LT in both hospital and societal perspectives (Supplementary Fig. 4).

**Fig. 1:**
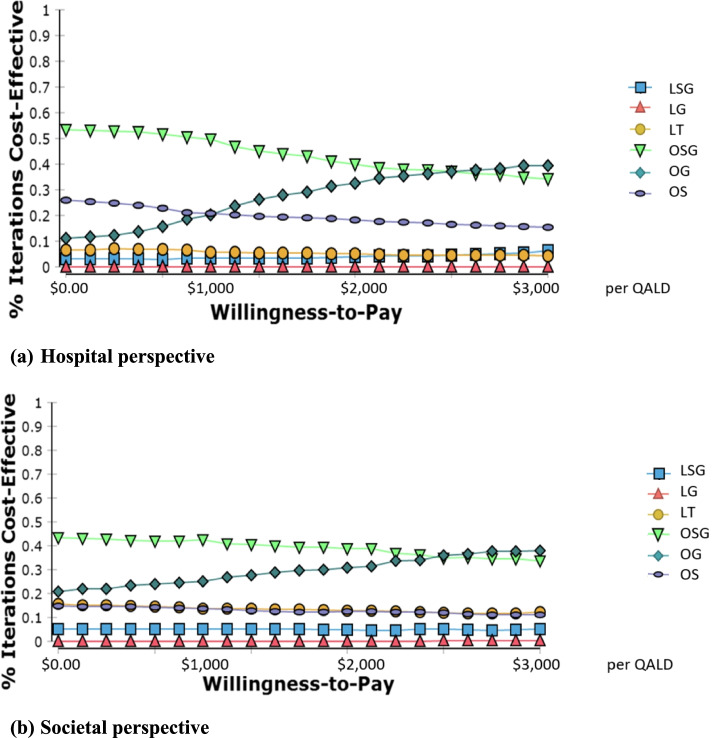
The cost-effectiveness acceptability curves (CEAC) of utility improvement LT = laparoscopic inguinal hernia repair using tacker, LG = laparoscopic inguinal hernia repair using glue, LSG = laparoscopic inguinal hernia repair using self-gripping mesh, OS = open inguinal hernia repair using suture, OG = open inguinal hernia repair using glue, OSG = open inguinal hernia repair using self-gripping mesh

The cost-effectiveness acceptability curves indicated that OSG dominated all other options, from both the hospital and societal perspectives, using thresholds ranging from $0 to $3,000. OG was the second most cost-effective option using a societal perspective across a wide range of thresholds and at higher thresholds using a hospital perspective, whereas OS was the second most cost-effective option at low thresholds using a hospital perspective (Fig. 1).

## Discussion

This prospective cohort study provided an opportunity to evaluate clinical and economic outcomes in 261 patients from data collected over 12 months. A total of 103 patients received LHR, with 47, 26, and 30 patients having tack, glue, and SGM mesh fixation, respectively; 158 patients received OHR with 117, 18, and 23 having suture, glue, and SGM mesh fixation, respectively. LSG had the highest hernia recurrence rate at approximately 10%, followed by OSG (8.70%), OG (5.56%), LG (3.85%), OS (3.42%), and LT (2.13%), respectively. OHR was associated with the highest rate of chronic groin pain, in particular OG.

Evaluation of economic outcomes identified higher overall costs associated with LHR than with OHR, with LSG and OS presenting the highest mean costs in each category. The mean overall utility scores calculated from multilevel mixed-effect linear regression models showed that LSG had the highest mean utility score of 0.975 (95% CI: 0.963, 0.987), followed by OS with mean utility of 0.973 (95% CI: 0.967, 0.979). The ICER, cost-effective acceptability curve, and cost-effectiveness planes suggested LSG was the least cost-effective in both utility outcomes and hernia recurrence prevention (i.e., higher costs and poorer outcomes). OSG had an acceptable cost-effectiveness plane for utility outcomes but was considered unacceptable for hernia recurrence prevention. OSG may be considered acceptable if the willingness to pay exceeded $2,500. The glue mesh fixation approach for both OHR and LHR presented an acceptable cost-effectiveness plane, with OG representing the best cost-effective acceptability curve for willingness to pay $0–2,500.

The international guideline for groin hernia management recommends mesh-based repair for symptomatic inguinal hernia patients, which can be either LHR or OHR [1]. The advantages of the former technique over the latter include less postoperative pain, [26, 27] paresthesia, [26] chronic groin pain, [28] more patient-reported satisfaction, [26] lower surgical wound complications, [27] shorter length of hospital stays, [29] earlier return to normal activities, [30] and fewer missed work hours [27]. However, many meta-analyses demonstrated no difference in recurrence rates between LHR and OHR [28, 31]. The current meta-analysis evidence of mesh fixation in OHR suggested that glue was superior to suture mesh at shorter operative time and reduced pain, but had no effect on long-term hernia recurrence, while chronic groin pain was equivocal. For SGM, no difference was found in the use of mesh fixation compared to suture or compared to flat mesh itself [9–19, 32–34]. In LHR, glue was more useful than tack in reducing chronic groin pain, but there was no difference in hernia recurrence [34–40]. There is no SGM information for LHR.

We conducted a prospective real-world cohort study to evaluate effectiveness in clinical practice [41] as a guideline for individual surgical decision-making in inguinal hernia repair [42]. In addition to knowledge, surgical preference, and skills, the surgeon's decision on the best mesh fixation for the patient will be aided by cost variables and economic evaluation [43]. Given the differences between randomized controlled trials and real-world data analysis and in particular the between-group heterogeneity, treatment-effect models were used for data analyses before performing the economic evaluation. The POMs and ATEs were evaluated by group, which had reasonably balanced covariable measures. Overlapping propensity scores indicated patients had similar chances of receiving the specified treatments. This study considered both hospital and societal perspectives between two time periods: short term (1-week postoperatively) and long-term (6 months postoperatively), and both utility and clinical (recurrence prevention) outcomes address hospital administration and patient decisions on the selection of inguinal hernia mesh fixation approaches.

Laparoscopic surgical costs are more expensive given the additional instrumentation requirements as compared with those required for open surgery. Mean utility scores from multiple mixed-effects linear regression estimates for both approaches were similar (0.939 compared to 0.972), with better laparoscopic mean utility scores for both glue and SGM compared to the corresponding open groups. OG mesh fixation was found to be the most cost effective, followed by OSG. From a utility point of view, the cost-effectiveness plane indicated that LG, OG, and OSG demonstrated better cost-utility than the conventional mesh fixation (tack in laparoscopic and suture in open repair). However, LG, OG, and OSG had poorer cost-effectiveness than conventional mesh fixation based on the prevention of hernia recurrence outcomes. A limitation of our approach was the high hernia recurrence rate; the adjustment was performed using available meta-analyses results. Our data suggest that LG, OG, and OSG were more cost effective than the conventional mesh fixation, with LSG being more effective but also more costly than LT. In light of the cost-effectiveness acceptability curves, OG demonstrated the highest probability of being the most cost effective, followed by OSG indicating non-penetrating mesh fixation (glue or SGM) can be an alternative mesh fixation to open surgery. As laparoscopic surgery is more costly, despite the improved cost-utility and cost-effectiveness of LG for inguinal hernia repair, the lower recurrence rate cannot sufficiently determine their willingness to pay.

Our literature review failed to find evidence of a previous economic evaluation of inguinal hernia mesh fixation approaches. The hernia recurrence rate is the outcome of highest concern, likely compounded by the surgical approach performed. Surgical techniques that can increase recurrence rates include lack of mesh overlap, improper mesh choice, and lack of proper mesh fixation. International guidelines for groin hernia management recommend the use of non-penetrating mesh fixation for hernia repair to reduce postoperative pain [1]. Previous comparisons between glue and tack mesh fixation in TEP suggested a hospital cost-saving benefit for glue based on reduced surgical operation time, postoperative hospitalization time, and lower rates of adverse outcomes [44]. According to international guideline management for groin hernias, SGM has proven controversial for open repair, whereas evaluation of laparoscopic approaches is limited due to lack of data [1]. As such, reduction in the cost of SGM and surgical equipment required for laparoscopic hernia repair, coupled with improved surgical approaches may make nonpenetrating mesh fixation a more viable alternative for laparoscopic hernia repair [45].

This research has limitations. The observational design and the population in each group was not balanced. We chose real-world data as we believe that each surgeon has a preferred inguinal hernia repair technique (open vs. laparoscopic and mesh fixation method). The surgeon's preference may be influenced by RCT protocol violations or the outcome. However, the inverse proportion treatment weight method was able to balance the group variables and make the groups comparable. Multilevel mixed-effects linear regression modelling was used to robustly identify the intervention effects.

## Conclusion

In conclusion, mesh fixation approaches for the treatment of inguinal hernia repair were not significantly different with respect to hernia recurrence rates and cost-effectiveness analyses. Glues and self-gripping mesh for open inguinal hernia repair were alternative approaches for mesh fixation based on a cost-utility analysis; however, this would be dependent on a willingness to pay.

## Supplementary Information

**Additional file 1: Supplement table 1.** Multilevel mixed-effects linear regression model comparing co-variables with mean utility score among 6 intervention groups.** Supplement table 2. **Describe balance of co-variables among 6 intervention groups: treatment multi-logit model.** Supplement table 3.** Comparisons of utility scores among 6 interventions: treatment-effect model.

**Additional file 2: Supplement figure 1.** Overlap plot LT = laparoscopic inguinal hernia repair using tacker, LG = laparoscopic inguinal hernia repair using glue, LSG = laparoscopic inguinal hernia repair using self-gripping mesh, OS = open inguinal hernia repair using suture, OG = open inguinal hernia repair using glue, OSG = open inguinal hernia repair using self-gripping mesh **Supplement figure 2.** Covariate balance density LT = laparoscopic inguinal hernia repair using tacker, LG = laparoscopic inguinal hernia repair using glue, LSG = laparoscopic inguinal hernia repair using self-gripping mesh, OS = open inguinal hernia repair using suture, OG = open inguinal hernia repair using glue, OSG = open inguinal hernia repair using self-gripping mesh.** Supplement figure 3.** Incremental cost-effectiveness (ICER) plane of utility improvement.** Supplement figure 4.** Incremental cost-effective (ICER) plane in hernia recurrence case prevented from cohort study

## Data Availability

The authors confirm that the data supporting the findings of this study are available within the article and its supplementary materials.

## Abbreviations

CEA: Cost-effectiveness analysis
CUA: Cost-utility analysis
ICER: Incremental cost-effectiveness ratio
LHR: Laparoscopic inguinal hernia repair
OHR: Open inguinal hernia repair
SGM: Self-gripping mesh
VAS: Visual analog score
SSI: Surgical site infection
BMI: Body mass index
EQ-VAS: European quality of life visual analog scale
EQ-5D-5L: European quality of life five dimension five levels
SD: Standard deviation
MD: Mean difference
CI: Confidence interval
POM: Potential outcome mean
ATEs: Average treatment effects
OS: Open inguinal hernia repair with suture mesh fixation
OG: Open inguinal hernia repair with glue mesh fixation
OSG: Open inguinal hernia repair with self-gripping mesh
LT: Laparoscopic inguinal hernia repair with tacker mesh fixation
LG: Laparoscopic inguinal hernia repair with glue mesh fixation
LSG: Laparoscopic inguinal hernia repair with self-gripping mesh
