# Supplementary material for: Cost-effectiveness analysis of mesh fixation techniques for laparoscopic and open inguinal hernia surgeries
Source: BMC Health Serv Res. 2022 Sep 6;22:1125. doi: 10.1186/s12913-022-08491-4 (PMC9450344; doi:10.1186/s12913-022-08491-4)
Supplement: Supplementary file 1 — Additional file 1: Supplement table 1. Multilevel mixed-effects linear regression model comparing co-variables with mean utility score among 6 intervention groups. Supplement table 2. Describe balance of co-variables among 6 intervention groups: treatment multi-logit model. Supplement table 3. Comparisons of utility scores among 6 interventions: treatment-effect model. [file 12913_2022_8491_MOESM1_ESM.docx]

**Supplement tables**

**Supplement table 1** Multilevel mixed-effects linear regression model comparing co-variables with mean utility score among 6 intervention groups.

**Supplement table 2** Describe balance of co-variables among 6 intervention groups: treatment multi-logit model.

**Supplement table 3** Comparisons of utility scores among 6 interventions: treatment-effect model.

**Supplement table 1** Multilevel mixed-effects linear regression model comparing co-variables with mean utility score among 6 intervention groups

|  | **Multilevel mixed-effects linear regression model** | | | | | |
| --- | --- | --- | --- | --- | --- | --- |
|  | **Coefficient** | **95% CI** | ***p-value*** | **Coefficient**  **legend** | **95% CI** | **Difference Utility to Reference** |
| LT | Reference |  |  | 0.965 | 0.955, 0.974 | Reference |
| LG | -0.019 | -0.033, -0.004 | 0.01 | 0.944 | 0.931, 0.957 | -0.021 |
| LSG | 0.007 | -0.007, 0.020 | 0.34 | 0.975 | 0.963, 0.987 | 0.010 |
| OS | 0.004 | -0.006, 0.015 | 0.40 | 0.973 | 0.967, 0.979 | Reference |
| OG | -0.021 | -0.037, -0.004 | 0.01 | 0.945 | 0.929, 0.960 | -0.028 |
| OSG | -0.026 | -0.041, -0.011 | 0.001 | 0.929 | 0.916, 0.943 | -0.043 |
| **Variables follow up time** |  |  |  |  |  |  |
| Follow up 1 week | 0.069 | 0.060, 0.078 | <0.001 |  |  |  |
| Follow up 1 month | 0.093 | 0.084, 0.102 | <0.001 |  |  |  |
| Follow up 6 months | 0.094 | 0.085, 0.103 | <0.001 |  |  |  |
| **Variables** |  |  |  |  |  |  |
| Age | -3.07e-06 | -0.00026, 0.00025 | 0.981 |  |  |  |
| Constipation | 0.011 | -0.001, 0.023 | 0.075 |  |  |  |
| Baseline utility | 0.191 | 0.147, 0.238 | <0.001 |  |  |  |

LT = laparoscopic inguinal hernia repair using tacker, LG = laparoscopic inguinal hernia repair using glue, LSG = laparoscopic inguinal hernia repair using self-gripping mesh, OS = open inguinal hernia repair using suture, OG = open inguinal hernia repair using glue, OSG = open inguinal hernia repair using self-gripping mesh

**Supplement table** **2** Describe balance of co-variables among 6 intervention groups: treatment multi-logit model

| Group | Co-variables | Standardized differences | | Variance ratio | |
| --- | --- | --- | --- | --- | --- |
|  |  | Raw | Weight | Raw | Weight |
| LG | Baseline utility | -0.274 | 0.044 | 1.240 | 0.697 |
| LSG | Baseline utility | 0.318 | 0.098 | 0.365 | 0.594 |
| OS | Baseline utility | 0.278 | 0.001 | 0.544 | 1.016 |
| OG | Baseline utility | -0.059 | -0.070 | 0.555 | 0.581 |
| OSG | Baseline utility | -0.534 | -0.098 | 1.753 | 0.837 |
| LG | Age | -0.059 | 0.026 | 1.128 | 1.277 |
| LSG | Age | 0.281 | -0.060 | 0.496 | 0.630 |
| OS | Age | 0.233 | 0.005 | 0.659 | 0.832 |
| OG | Age | -0.383 | 0.084 | 1.520 | 1.094 |
| OSG | Age | 0.266 | -0.035 | 0.566 | 0.775 |
| LSG | Constipation | -0.590 | -0.505 | 0.000 | 0.000 |
| LG | Constipation | 0.467 | 0.002 | 1.793 | 1.004 |
| OS | Constipation | -0.329 | -0.015 | 0.383 | 0.963 |
| OG | Constipation | 0.188 | 0.055 | 1.375 | 1.135 |
| OSG | Constipation | 0.183 | -0.066 | 1.361 | 0.841 |
| LG | Follow-up time | 0.000 | 0.001 | 1.004 | 0.999 |
| LSG | Follow-up time | 0.000 | -0.001 | 1.003 | 1.001 |
| OS | Follow-up time | -0.003 | -0.001 | 0.995 | 0.999 |
| OG | Follow-up time | 0.000 | 0.001 | 1.009 | 1.000 |
| OSG | Follow-up time | -0.015 | -0.010 | 0.997 | 0.984 |

LT = laparoscopic inguinal hernia repair using tacker, LG = laparoscopic inguinal hernia repair using glue, LSG = laparoscopic inguinal hernia repair using self-gripping mesh, OS = open inguinal hernia repair using suture, OG = open inguinal hernia repair using glue, OSG = open inguinal hernia repair using self-gripping mesh

**Supplement table 3** Comparisons of utility scores among 6 interventions: treatment-effect model.

|  | **Potential Outcome Mean (PoM)** | | | **Average Treatment Effect (ATE)** | | |
| --- | --- | --- | --- | --- | --- | --- |
|  | **coefficient** | **95% CI** | ***p-value*** | **coefficient** | **95% CI** | ***p-value*** |
| LT | 0.966 | 0.959, 0.973 | <0.0001 | reference |  |  |
| LG | 0.951 | 0.938, 0.964 | <0.0001 | -0.015 | -0.029, -0.0004 | 0.042 |
| LSG | 0.970 | 0.955, 0.984 | <0.0001 | 0.004 | -0.012, 0.019 | 0.608 |
| OS | 0.969 | 0.964, 0.975 | <0.0001 | 0.004 | -0.004, 0.011 | 0.377 |
| OG | 0.941 | 0.927, 0.955 | <0.0001 | -0.025 | -0.040, -0.010 | 0.001 |
| OSG | 0.932 | 0.913, 0.951 | <0.0001 | -0.034 | -0.054, -0.014 | 0.001 |

LT = laparoscopic inguinal hernia repair using tacker, LG = laparoscopic inguinal hernia repair using glue, LSG = laparoscopic inguinal hernia repair using self-gripping mesh, OS = open inguinal hernia repair using suture, OG = open inguinal hernia repair using glue, OSG = open inguinal hernia repair using self-gripping mesh
