# Supplementary material for: Cost-effectiveness analysis of mesh fixation techniques for laparoscopic and open inguinal hernia surgeries
Source: BMC Health Serv Res. 2022 Sep 6;22:1125. doi: 10.1186/s12913-022-08491-4 (PMC9450344; doi:10.1186/s12913-022-08491-4)
Supplement: Supplementary file 2 — Additional file 2: Supplement figure 1. Overlap plot LT = laparoscopic inguinal hernia repair using tacker, LG = laparoscopic inguinal hernia repair using glue, LSG = laparoscopic inguinal hernia repair using self-gripping mesh, OS = open inguinal hernia repair using suture, OG = open inguinal hernia repair using glue, OSG = open inguinal hernia repair using self-gripping mesh Supplement figure 2. Covariate balance density LT = laparoscopic inguinal hernia repair using tacker, LG = laparoscopic inguinal hernia repair using glue, LSG = laparoscopic inguinal hernia repair using self-gripping mesh, OS = open inguinal hernia repair using suture, OG = open inguinal hernia repair using glue, OSG = open inguinal hernia repair using self-gripping mesh. Supplement figure 3. Incremental cost-effectiveness (ICER) plane of utility improvement. Supplement figure 4. Incremental cost-effective (ICER) plane in hernia recurrence case prevented from cohort study [file 12913_2022_8491_MOESM2_ESM.docx]

**Supplement figures**

**Supplement figure 1** Overlap plot

**Supplement figure 2** Covariate balance density

**Supplement figure 3** Incremental cost-effectiveness (ICER) plane of utility improvement

**Supplement figure 4** Incremental cost-effective (ICER) plane in hernia recurrence case prevented from cohort study.


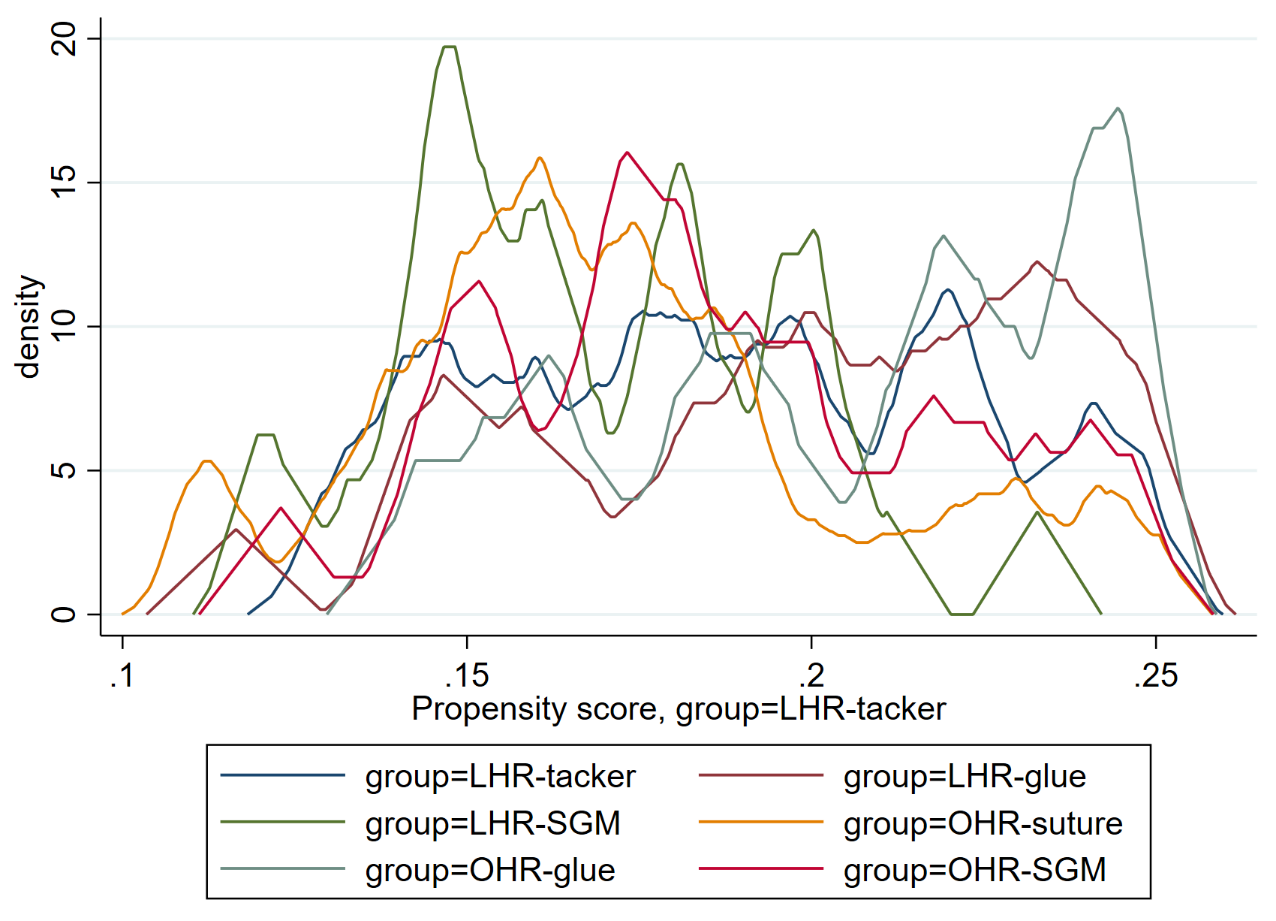


**Supplement figure 1** Overlap plot

LT = laparoscopic inguinal hernia repair using tacker, LG = laparoscopic inguinal hernia repair using glue, LSG = laparoscopic inguinal hernia repair using self-gripping mesh, OS = open inguinal hernia repair using suture, OG = open inguinal hernia repair using glue, OSG = open inguinal hernia repair using self-gripping mesh


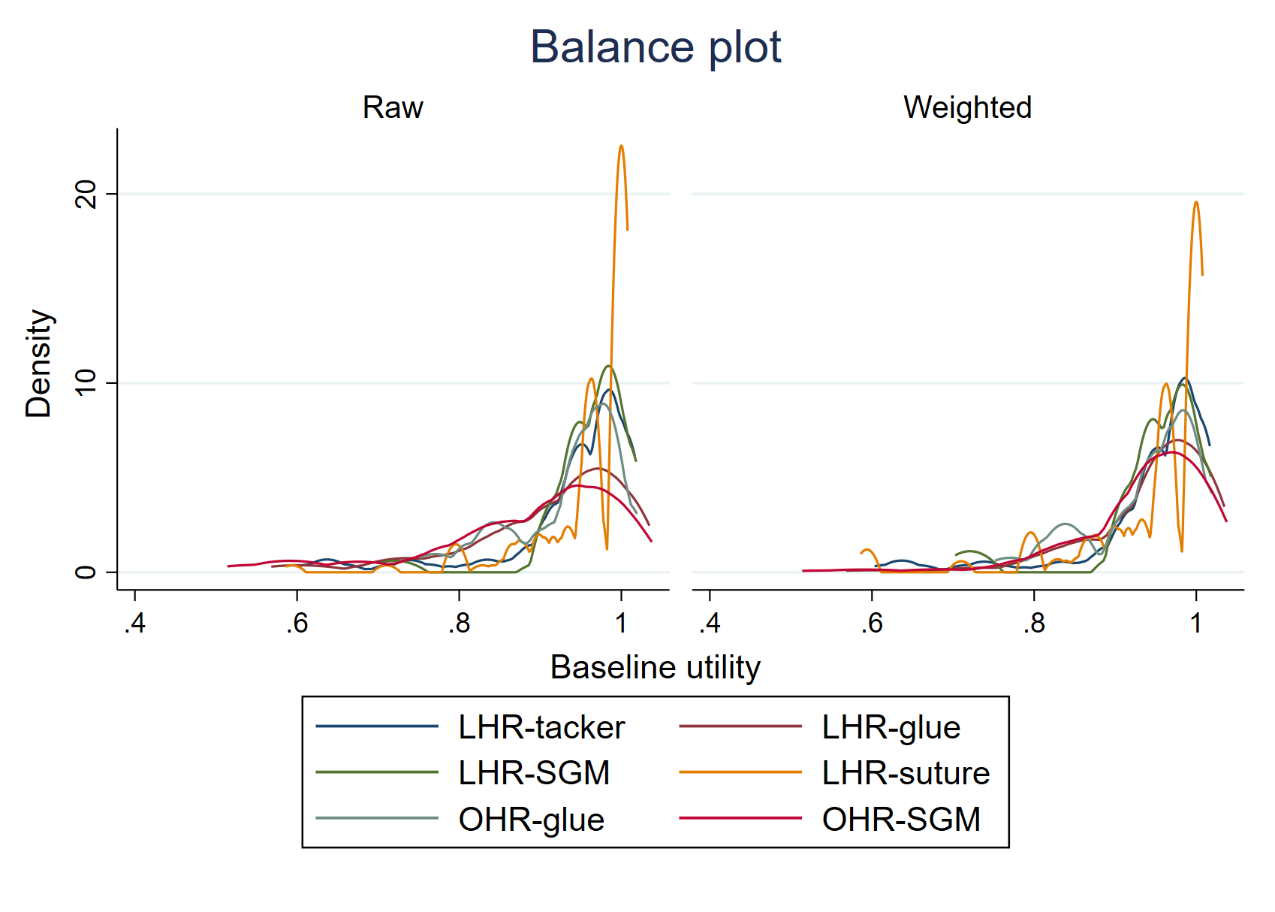


**Supplement figure 2** Covariate balance density

LT = laparoscopic inguinal hernia repair using tacker, LG = laparoscopic inguinal hernia repair using glue, LSG = laparoscopic inguinal hernia repair using self-gripping mesh, OS = open inguinal hernia repair using suture, OG = open inguinal hernia repair using glue, OSG = open inguinal hernia repair using self-gripping mesh

|    1. **Short term with hospital perspective** |
| --- |
|    1. **Long term with hospital perspective** |

|    1. **Short term with societal perspective**    |
| --- |
| 1. **Long term with societal perspective** |

**Supplement figure 3** Incremental cost-effectiveness (ICER) plane of utility improvement

|    1. **Hospital perspective** |
| --- |
|    1. **Societal perspective** |

**Supplement figure 4** Incremental cost-effective (ICER) plane in hernia recurrence case prevented from cohort study
